# The clinicopathological features and prognosis of primary pulmonary lymphoepithelioma-like carcinoma: A systematic review and meta-analysis

**DOI:** 10.1371/journal.pone.0240729

**Published:** 2020-10-16

**Authors:** Liansha Tang, Nan Chen, Wenbo He, Jian Zhou, Jinjue Zhang, Zhangyu Lin, Zihuai Wang, Jianqi Hao, Feng Lin

**Affiliations:** 1 Department of Thoracic Surgery, West China Hospital, Sichuan University, Chengdu, China; 2 West China School of Medicine, Sichuan University, Chengdu, China; 3 Department of Biotherapy, Cancer Center, West China Hospital of Sichuan University, Chengdu, China; Chang Gung Memorial Hospital at Linkou, TAIWAN

## Abstract

**Background:**

Primary pulmonary lymphoepithelioma-like carcinoma (PPLELC) was a sparse subtype of unclassified lung cancer. The clinicopathologic features, prognostic factors and multimodality treatment regimens of LELC remain inconclusive. We conducted this systematic review and meta-analysis to address this deficit in current knowledge.

**Methods:**

We searched PubMed, Embase, and Web of Science to filtrate studies investigating on clinical features and prognostic factors of LELC up to Sep 9^th^, 2020. Fixed and random effect models were generated to present the incorporated hazard ratios (HR) and odds ratios (OR) with 95% confidence intervals (CI). The quality and heterogeneity of the included studies were also evaluated carefully.

**Results:**

This systematic review and meta-analysis included 13 retrospective studies with a total of 1294 patients. The incidence of programmed cell death-ligand 1 (PD-L1) expression in PPLELC varied from 63.3% to 75.8%. Positive PD-L1 expression was more likely to be found in patients under 60 years old (OR = 2.16, 95%CI: 1.19–3.89, P = 0.01) and was associated with worse disease-free survival (DFS) compared with negative PD-L1 expression (HR = 2.99, 95%CI: 1.23–7.28, P = 0.02). The pooled results showed that stage was the prognostic factor for both overall survival (OS) and DFS. Moreover, a significantly better outcome of PPLELC was observed in men (HR = 0.56, 95%CI: 0.33–0.95, P = 0.03) and patients who received radiation (HR = 0.46, 95%CI: 0.22–0.96, P = 0.04).

**Conclusion:**

PD-L1 expression was high in PPLELC patients. It was significantly associated with age under 60 and the unfavorable DFS. Stage and gender could be the prognostic factor for OS. Radiation could be the effective therapy for PPLELC.

## Introduction

In accordance with the World Health Organization (WHO) histological classification in 2015, there is an extremely rare subtype in unclassified lung cancer named primary pulmonary lymphoepithelioma-like carcinoma (PPLELC) [1–3], which used to be classified into non-small cell lung cancer (NSCLC). It firstly aroused concern in 1987 when Begin et al. [4] reported it. In addition, it predominantly occurred in Southeast Asia [5, 6] with only sporadic cases reported in the West [7, 8].

A large part of PPLELC patients were diagnosed at early stage, therefore they were reported to confer a superior prognosis compared with other histologic types of NSCLC [7, 9–16], with approximately 107 months vs 13 months (median overall survival), respectively [7, 11]. However, the poor outcomes were found in advanced PPLELC patients at 24 months of median survival roughly [17]. Moreover, the prognostic factors described previously were still in dispute [11, 12]. In regard to the significant changes introduced by the American Joint Committee on Cancer (AJCC) Cancer Staging Manual eighth edition, further scrutiny is warranted to present the association between the risk factors and prognosis of PLELC, and it is also necessary to determine the distinct features from LELC to other lung subtypes. The feature of PPLELC was resembled in that of undifferentiated nasopharyngeal carcinoma (NPC). Both of them were related to Epstein–Barr virus (EBV) infection [5, 9, 18]. Due to the similar etiologic and pathologic characteristics with NPC, non-surgical treatment of PPLELC should exert a probable more defined effect.

On account of the rareness of this malignancy, the strategy of standard treatment for advanced PPLELC patients has not been established by clinical trials. Multimodality approach was considered to be effective. Recently, immunotherapy has deemed as a promising therapy modality improving survival and quality of life for metastatic or recurrent patients except for chemotherapy and radiotherapy [19]. Programmed cell death 1 ligand 1 (PD-L1), as an inhibitory NSCLC cells surface molecule, binding with programmed cell death 1 (PD-1) receptor to suppress T cell proliferation and activation [12, 20]. Obstruction of PD-1/PD-L1 pathway delineated a novel prospect for immunotherapy [12]. Some meta-analyses [15, 21–25] have investigated that PD-L1 expression was linked to adverse clinicopathological factors and gave the increase of death risk in NSCLC, whereas others drew the opposite conclusions [26, 27]. Besides, Chang et.al. [28] illuminated that PD-L1 expression proportion presented remarkably higher in PPLELC than other NSCLCs, further elucidating that virus-associated tumor cells have dominating PD-L1 expression. Nonetheless, this rare disease type lacks enough available data, and the relationship between PD-L1 expression and clinicopathological characters, and prognostic outcome of remained unclear.

Therefore, we performed a comprehensive systematic review and meta-analysis to evaluate the clinical and pathological features of PPLELC. Apart from that, we also aimed to shed a light on the relationship of PD-L1 expression with clinicopathological parameters and prognostic outcomes in PPLELC patients.

## Methods

### Search strategy

An intensive search for related studies from databases inception date to Sep 9^th^, 2020 was carried out on PubMed, Embase and Web of Science databases. There was no language restriction. We make the use of the search terms “lymphoepithelioma-like carcinoma*” OR “LELC*” AND “pulmonary*” OR “lung*” with related terms including MeSH terms as well as keywords. Our review was on the strength of the preferred guidelines for systematic reviews and meta-analyses [29]. All potentially relevant papers were searched and evaluated in detail. We also performed a recursive search for the bibliographies of all identified relevant literatures.

### Selection criteria

Articles were filtrated independently by two investigators (LS Tang AND N. Chen) utilizing predesigned eligibility forms, based on the predefined eligibility criteria. The third investigator (WB He) resolved the disagreements between investigators by consensus and arbitration. Eligible studies met the following criteria were considered to be included: 1) the articles included patients diagnosed with PPLELC by the criteria approved by the WHO [3]. 2) the studies investigated the clinical features or prognostic factors of PPLELC; 3) the relationship between PD-L1 expression and clinicopathological features and outcomes was revealed in the study concerning PD-L1. 4) the data regarding the clinicopathological features or prognostic information was sufficient, collective and available. 5) Hazard ratios (HR) with 95% confidence intervals (CI) were provided in the studies, or adequate information to assess them. The exclusion criteria were as follows: 1) meta-analyses, reviews, conference articles, in vivo or vitro studies, case reports, editorials, letters and expert opinions, non-English articles; 2) the data was absent and unavailable; 3) the sample size were less than 20 patients; 4) duplicated publications. In circumstances where there was over one publication attributed to the same patient cohort, we utilized the newest publication for analysis.

### Data extraction

Two investigators abstracted data from the eligible studies independently. Any discrepancies between two investigators was resolved by discussing with the third reviewer. The collected information was as follows: last name of first author, publication year, country, ethnicity, sample source, number of patients, tumor stage, treatment and follow-up time. Clinicopathological parameters data, PD-L1 expression and HR with 95% confidence CI for prognostic factors were also recorded.

### Statistical analysis

All statistical analysis was appraised by software STATA version 12.0. HR and 95% CI were generated from the clinical researches, which were used for accessing the relationship between prognostic features and overall survival (OS) or disease-free survival (DFS). Incorporative odds ratios (OR) with 95% CIs were also presented for dichotomous variables, which assessed the association between clinicopathologic features (age, smoking status, gender, pathologic stage) and PD-L1 expression. I^2^ and Q statistics were assessed as measures of heterogeneity. Depended on the degree of heterogeneity, random-effects models for conservative estimate favored if significant heterogeneity exists (I^2^>50%), otherwise, fixed-effects models were applied [30]. In order to assess the robustness of each outcome, sensitivity analysis was conducted by dropping out each included study. Egger’s rank correlation test and Begg’s weighted regression test were performed to estimate publication bias. Trim and fill procedure were conducted when publication bias exists.

## Results

### Search results and characteristics of studies

**Fig 1:**
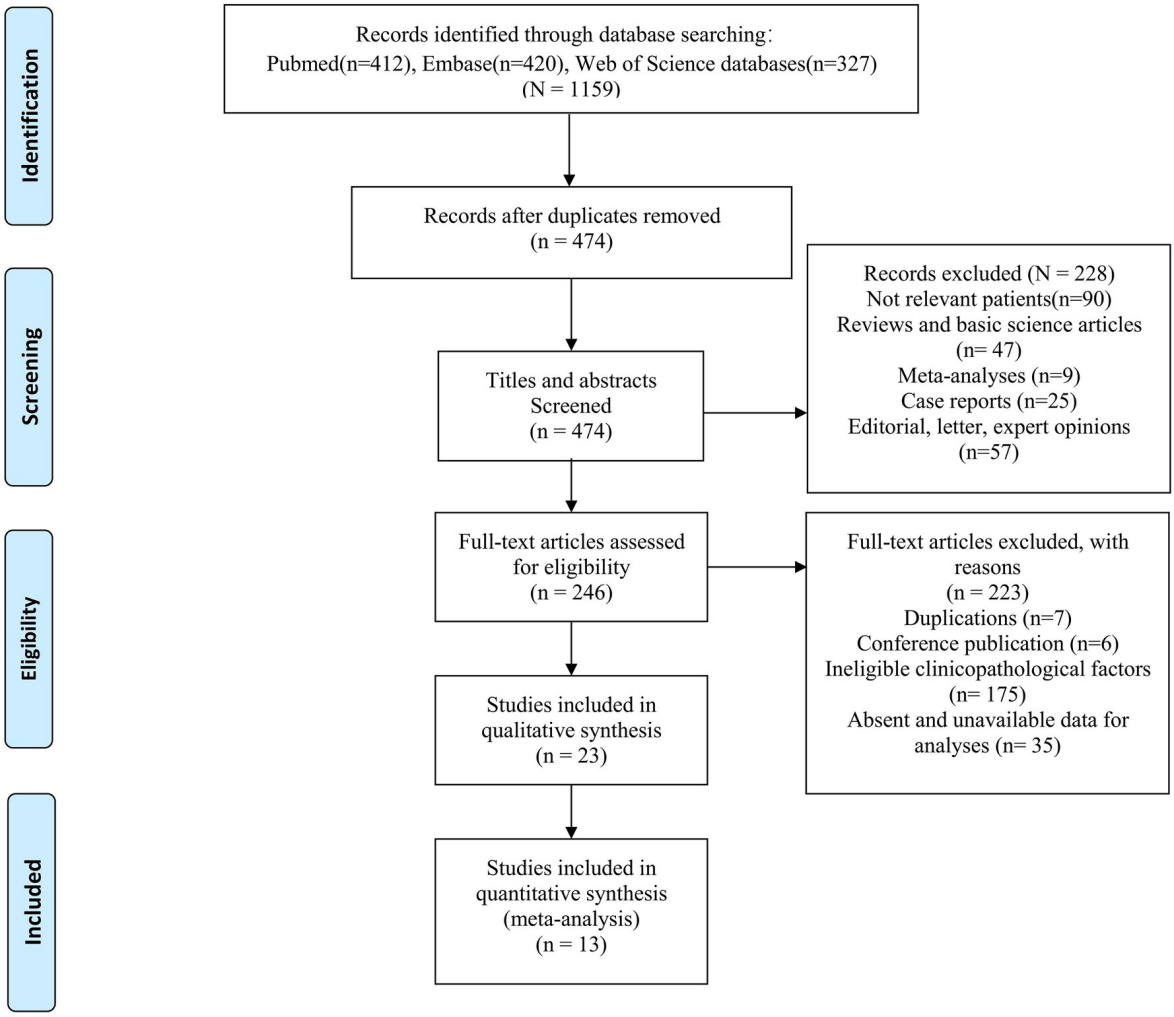
Flow diagram.

A total of 1159 potentially relevant studies were retrieved by the initial search strategies, including 412 in Pubmed, 420 in Embase, and 327 in Web of Science. Eventually, we identified 13 eligible studies of 1294 LELC patients for this meta-analysis after full text reading (Fig 1). Among these 13 studies, ten only included PPLELC patients [10, 11, 13, 14, 19, 28, 31–34], while three [12, 35, 36] compared PPLELC with other cancer subtypes (adenocarcinoma [12, 35, 36], squamous cell carcinoma(SCC) [12, 35, 36], neuroendocrine tumors(NET) [36], large cell carcinoma [12]. Except one study investigated patients in East, Northern Plains, Pacific Coast and Southwest based on the Surveillance, Epidemiology, and End Results database (SEER) [11], remaining studies were conducted on Asian population due to the highly prevalent proportion of PPLELC. However, patients were from the same center in five studies [10, 12, 13, 19, 34], we compared study population and patient characteristics in each study and found these were not identical. Therefore, we included all the parameters which could be incorporated. In addition, one study [14] included patients who only underwent surgery, one study [34] included patients who only received chemotherapy or radiation, the rest [10–13, 19, 28, 31–33, 35, 36] recruited patients who received multimodality treatment. The range of follow-up time varied from 26.6 mouths to 67 months (Table 1).

**Table 1:** Main characteristics of the included studies.

Author	Year	Country	Ethnicity	Cancer Type	N^a^		Treatment	Stage	Follow-up	NOS
					LELC	others				
**Chang**	2015	China	Asian	LELC	66		Surgery, Surgery+CT/RT/CRT	I/II/III/IV	NA	9
**Chen**	2019	China	Asian	LELC vs others	42	134	Surgery, Surgery+CT, CT/RT,CT+RT	I/II/III/IV	26.6 months	8
**Fang**	2015	China	Asian	LELC vs others	113	101	Surgery, Surgery+CT	I/II/III/IV	38.47mouths	9
**He**	2015	China	Mixed	LELC	62		Surgery, Surgery+RT	localized, regional and distant	67 mouths	8
**Jiang**	2015	China	Asian	LELC	79		Surgery, Surgery+CT/RT, CT	I/II/III/IV	35.02 mouths	9
**Jiang**	2016	China	Asian	LELC	43		Surgery, CT, RT	I/II/III/IV	30.5 mouths	8
**Lin**	2016	China	Asian	LELC	39		Surgery, Surgery+CT/RT/CRT	I/II/IIIA	26 mouths	8
**CY Lin**	2019	China	Asian	LELC	71		Surgery, Surgery+CT/RT/CRT	I/II/III/IV	34.1 months	7
**Z Lin**	2019	China	Asian	LELC	127		CT/RT	IIIB/IIIC/IV	22.7 months	8
**Xie**	2017	China	Asian	LELC	429		Surgery	I/II/III/IV	4.5 years	7
**Yu**	2018	China	Asian	LELC	87		Surgery, Surgery+CT/RT, CT	I/II/IIIA	34 mouths	8
**XY Yu**	2018	China	Asian	LELC	67		Surgery, Surgery+CT	I/II/IIIA	33 mouths	8
**Zhou**	2019	China	Asian	LELC vs others	69	1692	Surgery+CT/RT/CRT, CT/RT/CRT	I/II/III/IV	NA	8

^a^ Number of recruited patients.

LELC: Lymphoepithelioma-like carcinoma; CT: Chemotherapy; RT: Radiotherapy; CRT: Chemoradiotherapy; NA: Not available; OS: Overall survival; DFS: Disease-free survival.

### PD-L1 expression and clinical characters of PPLELC

**Fig 2:**
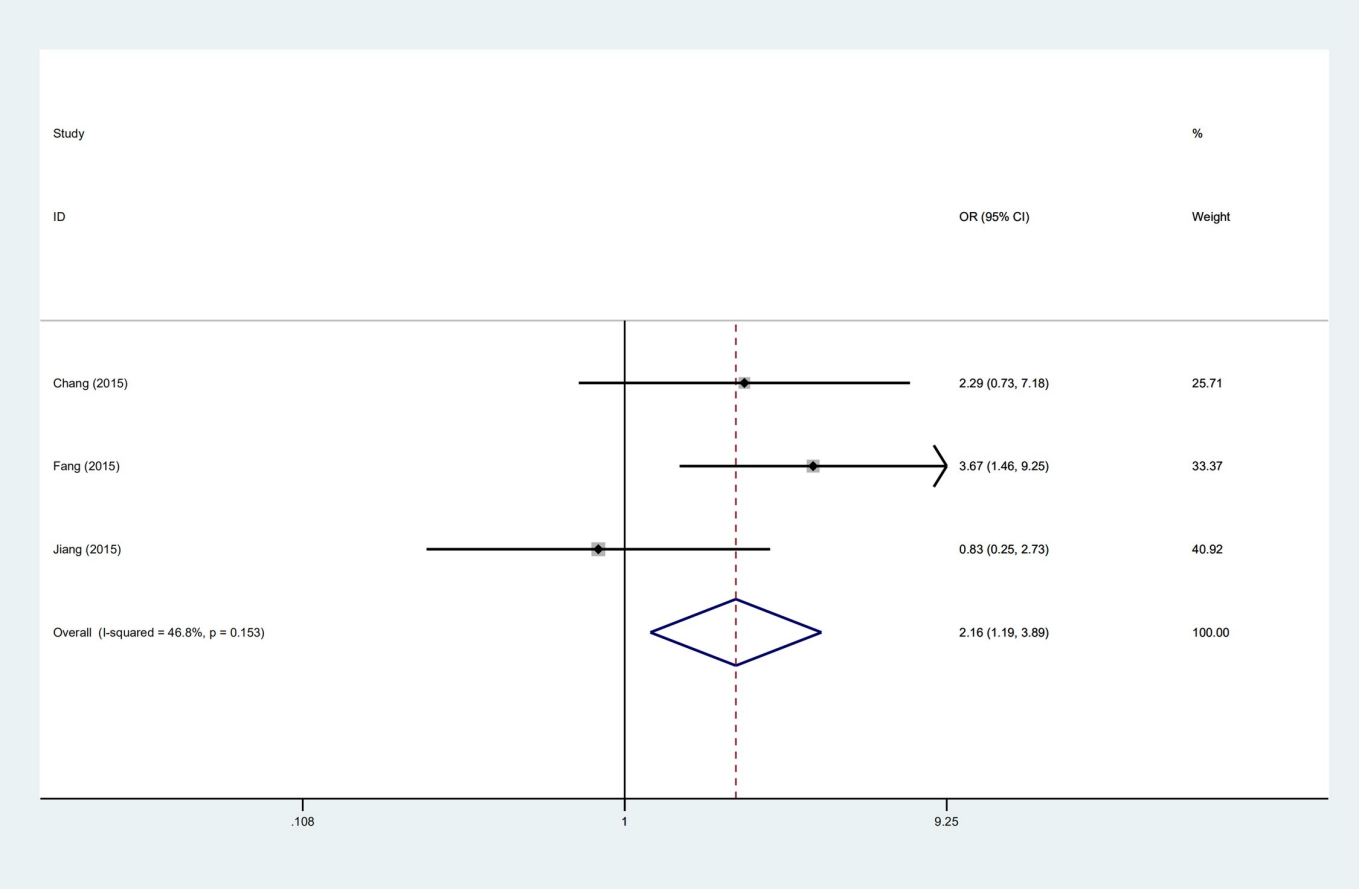
Forest plot describing the association between PD-L1 expression and clinicapathological features.

The expression of PD-L1 was detected by immunohistochemistry in four studies [12, 13, 19, 28]. Among these studies, the highest expression frequency reported by Chang et al. [28] was 75.8% while the lowest was 63.3% reported by Jiang et al. [13]. Three studies appraised the association of age with PD-L1 expression [12, 13, 28] and the incorporative consequence suggested that PD-L1 positive expression more tended to occur in patients under 60 years old (OR = 2.16, 95%CI: 1.19–3.89, P = 0.01) (Fig 2) and was associated with poor DFS (HR = 2.99, 95%CI: 1.23–7.28, P = 0.02) (Fig 4). However, gender (OR = 1.07, 95%CI: 0.66–1.73, P = 0.79), stage (OR = 1.45, 95%CI: 0.83–2.56, P = 0.20) and smoking status (OR = 0.59, 95% CI: 0.34–1.03, P = 0.06) were indicated no substantial association with PD-L1 expression (Table 2).

**Table 2:** Associations between clinicopathological features and PD-L1 expression.

Categories	Variables	N^a^	Pooled data			Heterogeneity
			OR/95%CI	P^b^	*I*^*2*^	*Ph*^c^
**Age**	*<60 vs≥60*	3	2.16 (1.19, 3.89)	**0.01**	46.8%	0.15
**Gender**	*Male vs female*	4	1.07 (0.66, 1.73)	0.79	0%	0.41
**Smoking**	*Ever vs never*	3	0.59 (0.34, 1.03)	0.06	0%	0.67
**Stage**	*I/II vs III/IV*	3	1.45 (0.83, 2.56)	0.20	0%	0.96

^a^ Numbers of studies included in the meta-analysis.

^b^ the significance of pooled OR.

^c^ the significance of heterogeneity test.

OR: Odds ratio; 95%CI: 95% confidence interval; *I*^*2*^: Value of *X*^*2*^ based I-squared statistics.

### Prognostic factors of PPLELC

**Fig 3:**
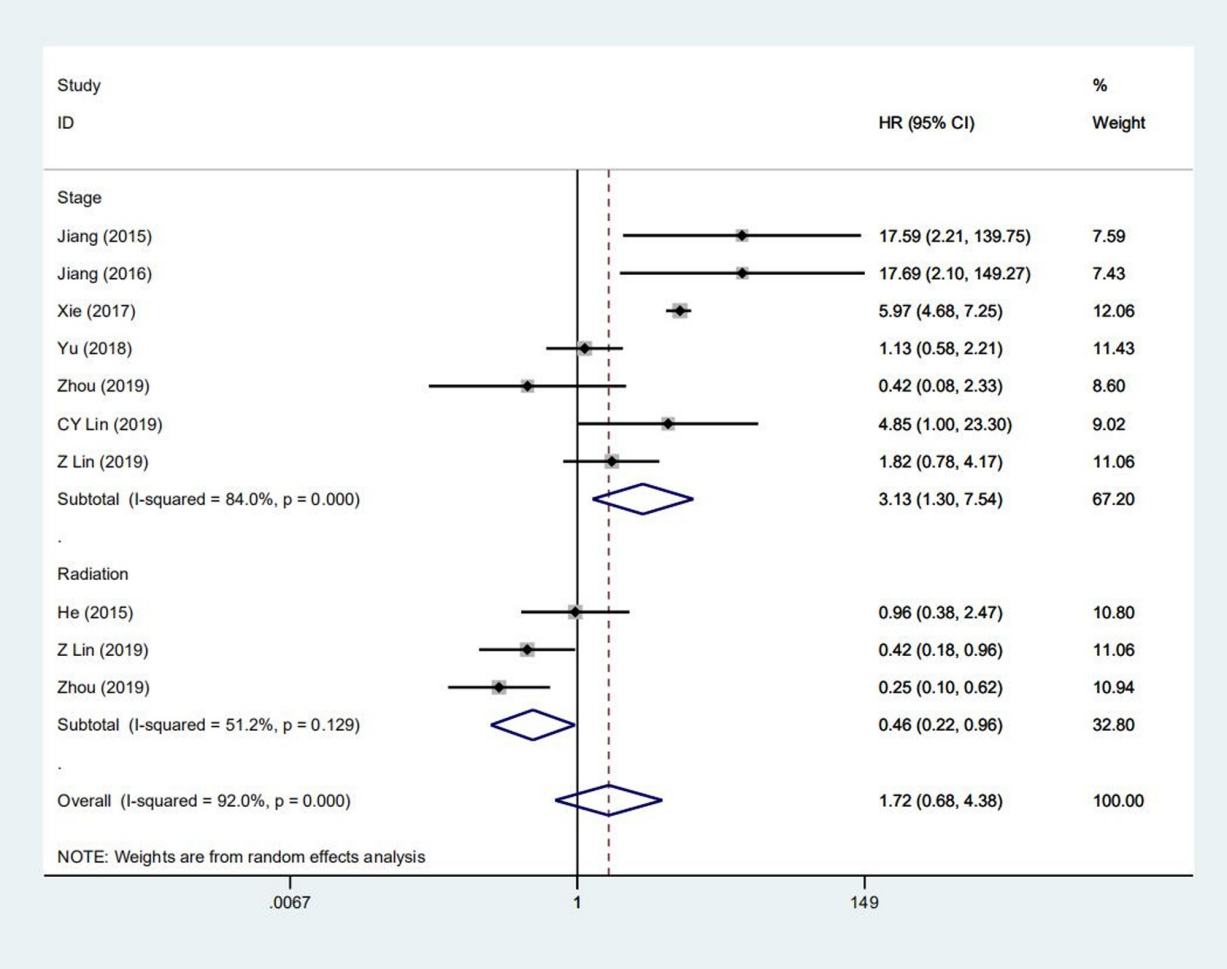
Forest plot describing subgroup analysis of the clinicapathological features and OS.

**Fig 4:**
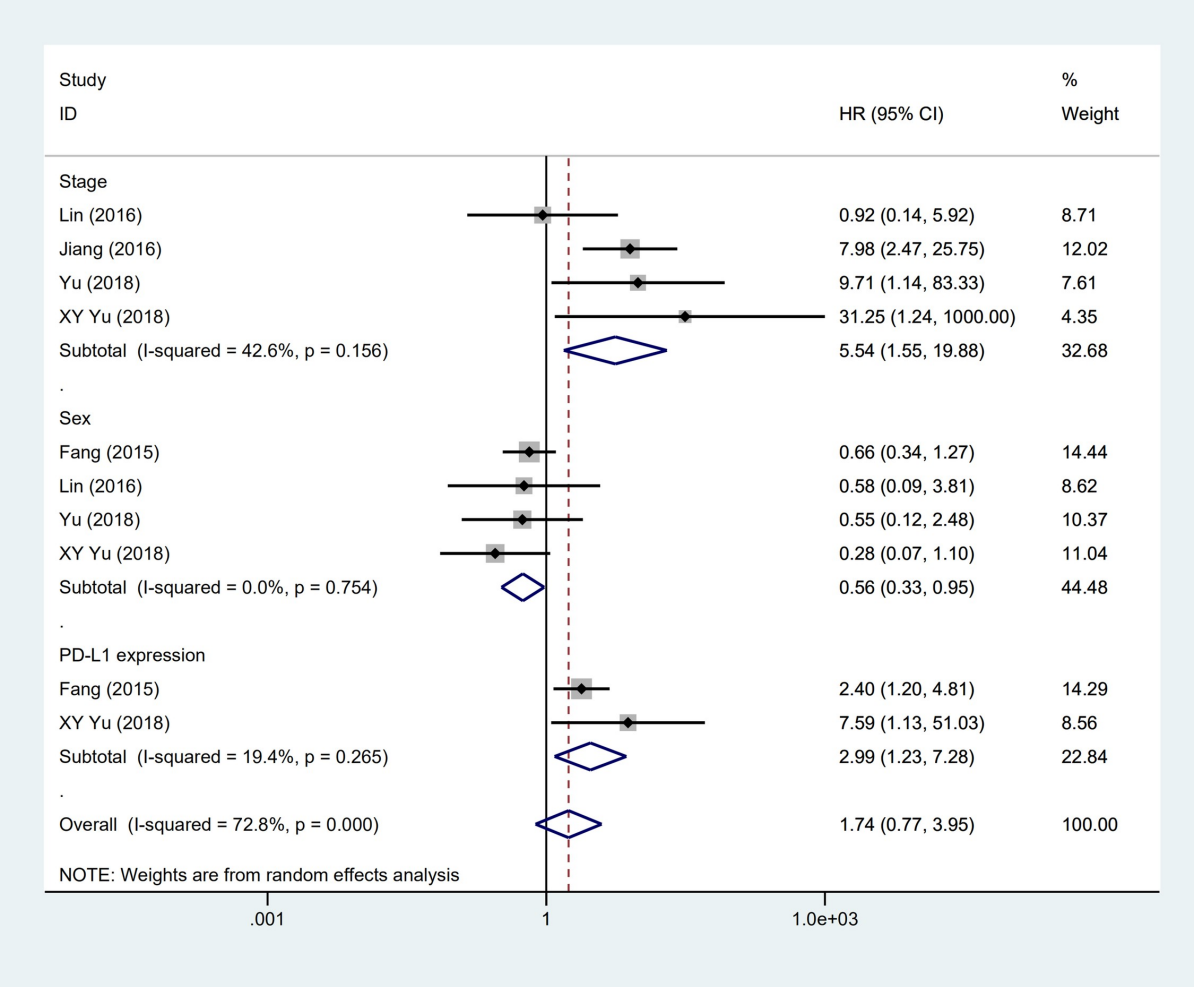
Forest plot describing subgroup analysis of the clinicapathological features and DFS.

Over 30 prognostic factors were analyzed in all 13 studies, however, only nine of them were further synthesized in meta-analysis because at least 2 studies mentioned (Table 3). Our outcomes are OS and DFS. As for OS, the pooled results demonstrated that PLELC patients underwent radiation were related to superior prognosis, with a 54% decreased risk of mortality (HR = 0.46, 95% CI:0.22–0.96, P = 0.04) (Fig 3). While in terms of DFS, patients with significantly better DFS was observed in male patients (HR = 0.56, 95% CI: 0.33–0.95, P = 0.03) (Fig 4). Besides, the incorporated results demonstrated stage was a significant prognostic factor for OS (HR = 3.13, 95%CI:1.30–7.54, P = 0.01) (Fig 3) and DFS (HR = 5.54, 95%CI:1.55–15.88, P = 0.00) of PLELC patients (Fig 4). Other prognostic factors, such as the expression of PD-L1 and adjuvant therapy, failed to significantly relate to better outcome (HR = 0.91, 95% CI: 0.10–8.12, P = 0.93; HR = 2.72, 95% CI: 0.49–15.12, P = 0.25, respectively). In addition, N grade, smoking status, tumor diameter didn’t significantly improve the prognosis of primary PLELC.

**Table 3:** Prognostic factors of LELC in predicting overall survival and disease-free survival.

Categories	Variables	N^a^	Pooled data		Heterogeneity	
			HR (95%CI)	P^b^	*I*^*2*^	*Ph*^c^
**Overall survival**						
**Stage**	*advanced vs early*	7	3.13(1.30,7.54)	**0.01**	84.00%	0.00
**N grade**	*advanced vs early*	4	1.76(0.86, 3.63)	0.12	68.50%	0.02
**Age**	*<60 vs ≥60*	6	0.77(0.49,1.22)	0.27	52.00%	0.06
**Gender**	*male vs female*	7	0.92(0.84,1.02)	0.11	0.0%	0.64
**Smoking**	*smoking vs no-smoking*	6	1.30(0.58,2.92)	0.52	74.00%	0.00
**Tumor diameter**	*≤3 vs >3*	3	0.83(0.18, 3.87)	0.82	55.30%	0.11
**PD-L1 expression**	*yes vs no*	2	0.91(0.10, 8.12)	0.93	81.50%	0.02
**Surgery**	*no vs yes*	3	1.32(0.16,10.91)	0.80	93.4%	0.00
**Radiation**	*yes vs no*	3	0.46(0.22,0.96)	**0.04**	51.2%	0.13
**Disease-free survival**						
**Stage**	*advanced vs early*	4	5.54(1.55, 15.88)	**0.00**	42.60%	0.16
**N grade**	*advanced vs early*	3	2.42(0.84, 7.00)	0.10	54.90%	0.11
**Age**	*<60 vs ≥60*	3	1.01(0.92, 1.10)	0.89	0.0%	0.58
**Gender**	*male vs female*	4	0.56(0.33, 0.95)	**0.03**	0.0%	0.75
**PD-L1 expression**	*yes vs no*	2	2.99(1.23, 7.28)	**0.02**	19.4%	0.27
**Tumor diameter**	*≤3 vs >3*	2	0.89(0.60, 1.32)	0.57	0.0%	0.53
**Adjuvant therapy**	*yes vs no*	2	2.72(0.49, 15.12)	0.25	9.20%	0.29

^a^ Numbers of studies included in the meta-analysis.

^b^ the significance of pooled HR.

^c^ the significance of heterogeneity test.

HR: Hazard ratio; 95%CI: 95% confidence interval; *I*^*2*^: Value of Higgins I-squared statistics.

### Publication bias and sensitivity analysis

**Fig 5:**
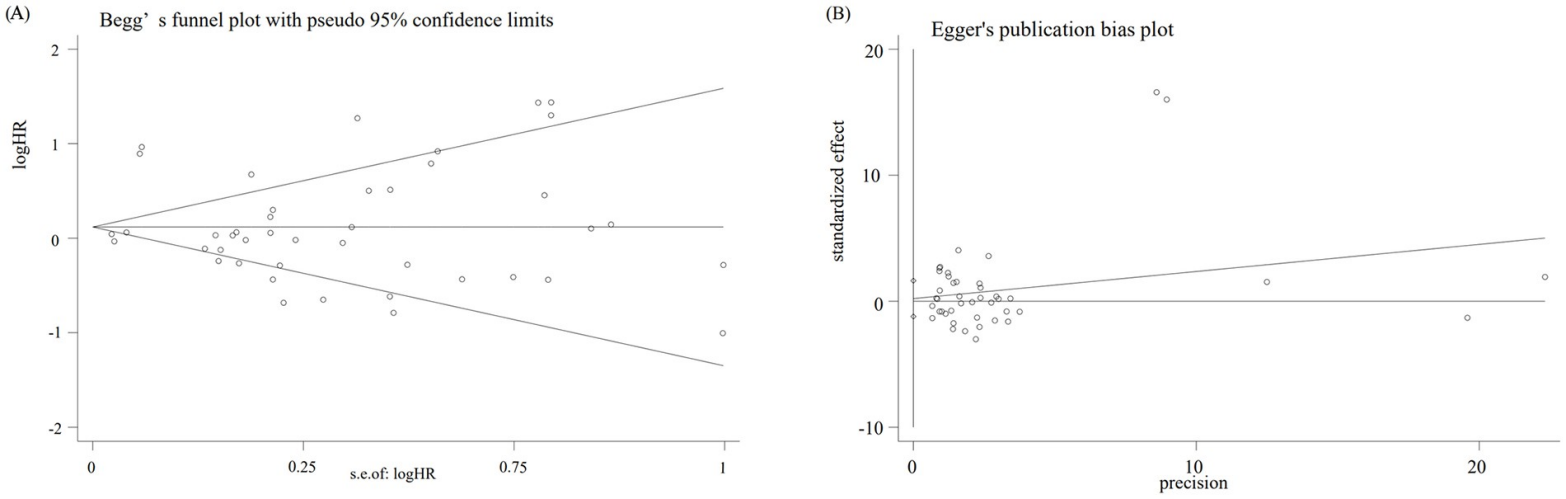
(A) Begg’s funnel plot with 95% confidence intervals for OS publication bias testing, (B) Egger’s funnel plot with 95% confidence intervals for OS publication bias testing.

Begg’s (P = 0.06) (Fig 5A) and Egger’s (P = 0.77) (Fig 5B) test presented no existence of publication bias among these included studies. At the meantime, the stability of this meta-analysis was validated by the sensitivity analysis with one study omitted. The final results defined no studies influenced the consequence, implying that the pooled results of the included studies were credible.

## Discussion

### Summary of evidence

Primary PLELC is an infrequent subtype of unclassified lung cancer. Whereas with regard to the clinicopathological features and prognostic factors of PPLELC, there still remained inconclusive. According to our analysis, patients under 60 years old were more possibly to show positive PD-L1 expression than those over 60 of PPLELC. Male and patients with negative PD-L1 expression showed promising DFS. Radiation was found to be effective for decreasing a 54% mortality risk in PPLELC patients. Stage was the prognostic parameter for both OS and DFS.

### Clinicalpathological and prognostic characters of PPLELC

LELC of lung had indistinguishable histology from undifferentiated NPC. The growth patterns were syncytial with heavy lymphocytic infiltration, while some tumor cells with low lymphocytic infiltration closely resembled non-keratinizing SCC. EBV-encoded RNA (EBER) detection could differentiate these two subtypes [37]. Except for that, several distinct morphologic characteristics were observed in PPLELC including spread through alveolar spaces, lepidic spreading pattern, focal keratinization and granulomatous inflammation [37].

In previous studies, PPLELC primarily occurred in young individuals and non-smokers without sexual predilection [10, 32, 38, 39]. The proportions reported by Qin et al. [38] were 71.8% and 72.9%, respectively. More specifically, the average age of PPLELC patients was generally younger than that of NSCLC patients [32]. The youngest case reported was 8 years old [40]. Different studies had different age predilection, much older was reported as well [11, 41]. A large cohort study from SEER database [11] found that the range of age was from 15 to 86 (median age:65 years old) at diagnosis, and majority of PPLELC patients were the male (58.1%, 36/62) and the white (64.4%, 40/62). Other clinical and pathological characteristics such as elevated EBV concentration, represented large tumor volume and more advanced stage in PPLELC patients [42]. Moreover, circulating EBV DNA might be positively related to tumor burden [43]. And our analysis included some comparative studies [35, 36] to find the unique features of PPLELC as well. PPLELC was prone to happen in middle age female compared with other histologic NSCLC [35, 36]. By comparison with SCC [35], the results showed that the diameter of LELC was smaller and computed tomography (CT) scans presented scarce vascular convergence signs and spiculation. Zhou et al. [36] reported that LELC subgroup was mostly originated from left lower lobe (29.0%) and right middle lobe (24.6%) but seldom observed in the right upper lobe(4.3%) compared with SCC and NET.

### The PD-L1 expression in PPLELC

PD-L1 was regarded positive by immunostaining (membranous staining>5%) usually [12, 13]. Furthermore, the proportion ranged from 5% to 49% and≥50% were defined as low and high PD-L1 expression respectively [44]. Multiplying the percent of positive staining cells by the intensity score determined semi-quantitative H score [12]. The high expression could also be defined as the cut-off value over 30 [12]. PD-L1 over-expression had 9 times higher chance to present in PPLELC than non-LELC (P = 0.028) [12]. Our merged data found that the incidence rate of PD-L1 expression varied from 63.3% to 75.8% in PPLELC. Besides, the relationship between PD-L1 expression and gene mutations of lung was also arouse concern in recent studies [25, 44, 45]. Unlike other NSCLC subtypes, PPLELC patients showed lower mutation rates of some common gene, such as ALK, KRAS and EGFR [25, 44, 45]. The explanation for this may be that there had a unique gene profile in LELC of lung [45]. Recently, Xie et al. [44] recruited 29 PPLELC patients in China to explore the genomic landscape. The results revealed the epigenetic regulators mutations enrichment, which indicated that the development of PPLELC may include chromatin modification and remodeling. They pointed that about half patients had at least one copy number variation predominated by CCND1 amplifications (30%, 8/27). Another study [46] revealed that the genetic features of PPLELC were clearly distinct from other NSCLC. Frequent absence of type I interferon genes and predominant signature 2 mutations(C>G and C>T mutations) were found in PPLELC. Some abnormal signaling pathways, such as JAK/STAT, NF-κB and cell cycle, were influenced by genetic enrichment.

In terms of other clinical and pathological factors, the relationship with PD-L1 expression was reported in some studies as well [11, 12, 19, 25]. T stage [12] and p53 expression [19] were reported to substantially associate with PD-L1 expression in PPLELC. A meta-analysis [25] included fifty studies of 11,383 patients showed that PD-L1 expression was more likely to present in smokers, male, and patients with large tumor size, poor tumor differentiation, and/or lymph nodal metastasis in NSCLC. However, no significant association was indicated between PD-L1 expression and age. Our analysis focused on this rare histologic subtype showed that positive expression was substantial linked to age in PPLELC, indicating patients under 60 years old may had more obvious correlation, thus it can be seen that these patients might have better treatment efficacy with the PD-1/PD-L1 pathway blocked. However, our results could not confirm the link between PD-L1 expression and other clinicopathological features such as sex, stage and smoking status. The reason may be as follows. Firstly, only two assays investigated the parameters with PD-L1 expression [12, 13]. Secondly, the different thresholds and detection technique of PD-L1 expression could contribute to this variability, therefore more large multicenter studies utilizing the unified cutoffs and antibody of PD-L1 expression are needed to acquire more precise results.

Additionally, the prognostic significance of PD-L1 expression have not arrived at any agreement yet. High PD-L1 expression was confirmed to suffer impaired DFS significantly [12, 19], while these studies [12, 13] showed no significant association between PD-L1 expression and OS. Jiang et al. [13] clarified that a longer progression-free survival(PFS) was substantially related to positive PD-L1 expression (HR = 3.86, 95%CI:1.41–10.59, P = 0.01). Relevant possible explanations about PD-L1 expression and the outcome of PPLELC were that unextinguished tumor cells with high PD-L1 expression reject immune elimination and the immune response cells are inhibited, which made the function of T cells down-regulated and promotes tumor development [47]. However, intensive lymphocytic infiltrate was confirmed to be the characteristic of PPLELC [13], which was generally recognized to be related to improved outcome [48, 49]. Our results found the significant association of PD-L1 expression with DFS rather than OS, thus we need more studies with more mature data to investigate the prognostic value of PD-L1 expression.

### The prognosis of PPLELC

Numerous researches have studied the prognostic factors of PPLELC. Early pathological stage [19], total metabolic tumor volume(MTV>72.6ml) [33], normal monocyte-to-lymphocyte ratio (MLR) [50], lactate dehydrogenase (LDH) [7] and serum albumin level [7] were reported to significantly associate with better OS of PPLELC. Our merged results presented that stage was the prognostic characteristic for OS and DFS for PPLELC. Theoretically, tumor in advanced stage would present impaired prognosis than that in early stage. However, staging has not been verified its independent predictive value [11]. Sensitivity to following therapy may influence the ultimate survival of the cancer, alternatively LELCs might need a specific staging system instead of one dedicated to lung cancer [36]. Except for that, patients using 18F-FDG PET for staging tend to have a superior OS (P = 0.003) [51]. Patients staged with 18F-FDG PET would have an improvement of 5-year OS from 49.7% to 85.4% (P = 0.012) [51]. In addition, positive serum EBV-DNA was reported to be the independent predictor of PFS [35]. In our meta-analysis, male patients were prone to have a superior DFS. This may result from the relatively low proportion of male in the included studies [10, 12, 19, 32], and female were considered to be predominated in PPLELC [35, 36].

The outcome of PPLELC were widely thought to be considerable. Qin et al. [38] reported that the 3-year OS for early stage patients reached 100%, and the 1-year and 2-year OS for advanced patients just decreased by 7% and 23%, separately. And in these two studies only recruited advanced PPLELC patients [34, 36], the median OS and PFS were 36.7 months and 7.7 months, respectively [36]. After receiving multimodality therapy, the disease-control and overall response rates for PPLELC patients were 80.6% and 61.8% [34].

### The multimodality therapy of PLELC

The multimodality treatment for PPLELC was universally recommended, but the existing handful of studies have not succeeded in setting a therapy standard on account of small simple size and the heterogeneous management of anti-tumor treatments. Several prior studies [11, 32, 36] demonstrated that complete resection surgery is the effective therapy method for early stage patients with pulmonary LELC. Patients received complete resection were reported to harbor remarkable 5-year survival [7, 32]. According to the highest metastatic frequencies investigated by Yu et al. [10], the invaded lymph nodes were most commonly involved in the right lower lobe (#7, 42.9%) and left lower lobe (#7, 40.7%), manifesting that precise resection may need the complete extent of lymph nodes dissection. However, radical surgery may not exert a great deal of influence on patients in higher MLR level [50] which presented a distinct higher relapse outcome compared with those in lower MLR level (5-year relapse rate: 40% vs 14%, P<0.05). Our results did not present the significant correlation between surgery and OS of pulmonary LELC, since most of the included studies showed various surgical approaches and enrolled early stage LELC patients. While Zhou et al. [36] recruited advanced stage LELCs, suggesting that sensitivity to chemoradiation might cloud the final outcome of this disease.

In terms of multimodality treatment, platinum-based doublets regimen, including paclitaxel/docetaxel (TAX/DOC) plus cisplatin/carboplatin (DDP/CBP), pemetrexed (PEM) plus DDP and so on, were regarded as the recommended therapy for PPLELC [41, 52, 53]. Recently, these studies compared the efficacy in different chemotherapy (CT) regimens [34, 36]. No significant differences were found among patients received taxane-based combinations or non taxane-based therapy in survival or response [36]. A real-world study included 127 patients with unresectable PPLELC by Lin et al. [34] reported that gemcitabine plus platinum (GP) achieved the longest PFS(GP vs AP vs TP:8.8 months, 6.4 months, 7.9 months; P = 0.031)and the best response rate(GP vs AP vs TP:63.2%, 21.1%, 30.0%; P = 0.005) by comparison with pemetrexed plus platinum (AP) and taxanes plus platinum (TP).

Radiotherapy was reported to be sensitive for PPLELC [34, 41]. And patients who combined chemotherapy had remarkable more favorable PFS and response [34]. In accordance with the clinical practice, we also recommended inducted chemotherapy before RT [34]. Our data suggested that radiotherapy could improve OS of PPLELC patients, which might be attributed to the similar biological characteristics with NPC. However, He et al. [11] failed to illuminate that radiation brought benefit to PPLELC patients in OS. In their study, white patients occupied for the biggest percentage (64.4%), and the author did not exclude the patients with multiple primary cancer. Above mentioned may influence the final results. Last but not least, the optimal approach and dose of RT should be well determined by some well-organized clinical studies in the future.

The immunotherapy and target therapy were considered to be promising approach for promoting the OS and cure rate of patients with resectable lesions of PPLELC. This promise was on the basis of marked efficacy of checkpoint inhibitors in NSCLCs [54, 55]. Few case reports have tested the efficacy of checkpoint inhibitors in LELC, including Nivolumab [17, 56, 57] and Pembrolizumab [36]. The majority of cases [17, 36, 56] got partial response from these newly inhibitors, while one patient was related to rapid progression [57]. Additionally, rare gene mutation (EGFR, ALK) rate in LELC might suggest the low sensitivity of TKIs target therapy [44]. TP53 mutation and some altered critical pathways were found to compromise the efficacy of TKI treatment in PPLELC [46] as well. Therefore, further studies conducted in a prospective or multicenter manner are indispensable to determine the treatment protocol.

### Strengths and limitations

There still have several limitations in present study which should be remarked. Firstly, on account of the relatively small sample size of PPLELC studies, we did not conduct the subgroup analysis, the merged results may have less significant statistical power as well. As a result, it’s essential to perform extra elaborated-designed original studies with larger population to obtain more integrated analysis of the clinicopathological and prognostic factor in patients with PPLELC. Second, distinct intrinsic discrepancy within patient populations couldn’t be ignored due to the Asian origin of the vast majority of patients included. Moreover, various patient selection criteria and different cut-offs of clinical and pathological features may hamper our pooled results. Additionally, our study included five studies from the same institution, which may influence the results of meta-analysis. However, we could not exclude the duplicate patients completely since the inclusion and exclusion criteria was not exactly the same. The overlapping might not be avoidable. However, we performed the sensitivity analysis and publication bias to assess the potential source of heterogeneity and no significant results were found. In spite of the limitations mentioned above, to our knowledge, this meta-analysis presents the first study to systematically assess the clinicopathologic features and prognostic factors of PPLELC. To strengthen our findings, profound large sample size studies are essential for analyzing this issue.

## Conclusion

PD-L1 expression was high in PPLELC patients and significantly associated with those under 60 years old. It was associated with the unfavorable DFS of pulmonary LELC. Stage and gender could be the prognostic factor for OS. Radiation could be the effective therapy for PPLELC.

## Supporting information

S1 File PRISMA 2009 checklist. (DOC)

S2 File (XLSX)
